# Age-Related Changes in the Clustering of Blood Populations in Cynomolgus Monkeys Depend on Sex and Immune Status

**DOI:** 10.3390/life13020316

**Published:** 2023-01-23

**Authors:** Dzhina D. Karal-ogly, Alexander N. Shumeev, Viktoria V. Keburiya, Marina V. Mintel, Stanislav A. Rybtsov

**Affiliations:** 1 The Research Institute of Medical Primatology, 354383 Sochi, Russia; 2 Centre for Cell Technology and Immunology, Sirius University of Science and Technology, 354340 Sochi, Russia; 3 Centre for Regenerative Medicine, University of Edinburgh, Edinburgh EH8 9YL, UK

**Keywords:** cynomolgus monkeys, *Macaca fascicularis*, as crab-eating macaques, aging, blood cells, non-human primates, sexual dimorphism, non-anthropoid primates, old world monkeys

## Abstract

Non-anthropoid primates cynomolgus monkeys (*Macaca fascicularis*), also known as crab-eating macaques, are increasingly used in biomedical and preclinical studies due to their evolutionary proximity to humans, sharing similar diets, infectious and senile diseases. Age-related changes and sexual dimorphism of the immune system of C. monkeys have not been sufficiently characterized in literature, though age and sex differences affect the course of diseases and sensitivity to medications. Aging in C. monkeys is accompanied by an increase in CD3+CD4+CD8+ (DP-T) cells, plasma B-cells, and a decrease in platelets. Erythromyeloid bias has also been noticed in older animals. There was an increase in eosinophils, haematocrit (HCT) and haemoglobin concentration (HGB). Senile decline in the function of the immune system had sex differences. An increase in the number of monocytes, cytotoxic lymphocytes (CTL) and a decrease in the T-helper population were more pronounced in older females. A significant reduction in the number of B-cells and activated T-cells was detected in males only. A moderate correlation with the regression model of aging was established for DP-T, HCT and HGB. The reduction in the B cells count in males and the increase in CTL level in females are moderately correlated with age. Other blood cell populations did not show significant correlations in the regression models due to their high sample variability. The novel cell population CD3-CD20loCD16/CD56+, presumably NK-cells subset, was revealed. This cell population demonstrated an increase trend with age in both sexes. Population-statistical age norms for different sexes for young and very old macaques were established. The blood population clusters associated with sex and immune status in older animals were also identified.

## 1. Introduction

In the aging human population, both the number of age-related pathologies and the burden on the healthcare system are growing. Analysis of the hematopoietic system, including aging markers, allows identifying cell populations that change quantitatively with age and analyse their function. Age-related changes in the hematopoietic system and immunity are often the cause of senile diseases [1,2]. Studies in mice, that are evolutionarily distant from humans provide important but insufficiently accurate information in preclinical drug trials [3]. Studying the age dynamics of cell populations and differences in cell markers in evolutionarily related species provides important insights into healthy and pathological aging. The research on anthropoid primates requires strong scientific and ethical justifications. On the contrary, testing in non-anthropoid primates (NAPs) is much easier to justify ethically for a wide range of important biological and preclinical research [4]. However, approaches to the study NAPs of old-world monkeys are faced with the lack of a comprehensive description of the haematopoiesis and the immune cells for animals of different ages. Such studies were carried out on only a few species and described a limited range of parameters [5,6].

In addition, due to the cost of long-term maintenance, it is extremely difficult to form a group of aged animals whose body condition would be comparable to humans in pre- and post-retirement age, i.e., group of people, that often shows deviations in their healthy aging, capable of realizing the potential to improve health, prolong productivity, and reduce future healthcare burdens.

Cynomolgus monkey (*Macaca fascicularis*) are evolutionarily close to *Homo sapiens*. They possess similar physiological and immunological characteristics and are widely used in preclinical studies. Similar to humans, C. macaques are sexually dimorphic in size and weight, and in blood chemistry [6]. While in their natural wild habitats, cynomolgus monkeys have pronounced seasonal breeding cycles, they also breed in other seasons in captivity. Sexual maturity occurs at four years of age in both females and males [7]. Similar to humans, they also have a monthly menstrual cycle (28–32 days), their fertility declines with age and the menopause occurs at the old age [8]. The life expectancy of the C. monkeys in captivity ranges from 25 to 30 years [6].

Although their traditional name is crab-eating macaques, they are omnivores similar to humans (able to digest a variety of foods). Their low weight (2.5–4.5 kg females and 4–8 kg males) allows reducing the expenses for breeding animals and reducing the volume and cost of the tested substances. Thus, this species is an excellent model for studying human metabolic diseases, as well as the microflora and pathologies of the intestine [9]. C. monkeys live longer in captivity than in the wild and, such as humans, are susceptible to age-related pathologies, including inflammatory, neurodegenerative disorders and pathologies of the cardiovascular system. They also show human-like changes in biochemical parameters, cognitive decline, osteoporosis, and arthritis [5,10].

C. macaques are predisposed to dangerous human viral diseases, including influenza virus, hepatitis A, Ebola-related virus, measles, monkeypox, herpes B, HIV-like disease, and coronaviruses due to high protein similarity of virus entry receptors with humans [11]. Moreover, the resistance of these animals to infections decreases with age, which suggests the similarities with humans in the aging of the immune system [12]. Further comparative analysis of the main blood populations, their age and sex related differences in the cynomolgus macaque and humans will provide a more detailed understanding of the similarities in the aging of the immune system of these two species, as well as determine which indicators can be used in preclinical trials.

The aim of this study was to determine the age dynamics of blood cell populations in *M. fascicularis* and to identify populations that change with age, as well as to discuss the similarities and differences in the aging of the *M. fascicularis* immune system with human aging. This study included a sample of old animals (17–31 years old). The presented parameters of the hematopoietic system of C. Macaque of such an advanced age have not been previously studied.

## 2. Methods

### 2.1. Husbandry of Animals and Sampling

**Figure 1:**
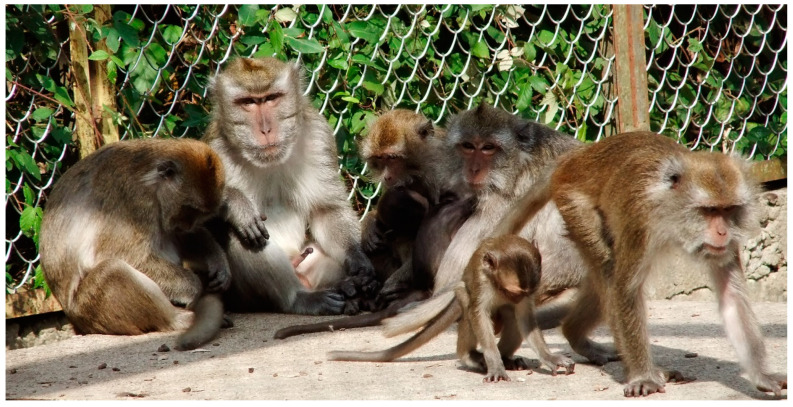
Family of cynomolgus monkeys *(Macaca fascicularis)* in the apery of the Research Institute of Medical Primatology (Sochi, Russia).

Four age cohorts of healthy *M. fascicularis* were assembled for research, each cohort included males and females. Group 1 (40 animals, 3–7 years old), Group 2 (23 animals, 17–31 years old). All animals were kept in the apery of Research Institute of Medical Primatology, Sochi, Russia, (Figure 1). Animals were kept in individual or family cages, at the ambient temperature (21–28 °C); relative humidity 40–70%; natural daylight hours. Sanitary measures were carried out in accordance with local law. Access to fresh water for animals was provided ad libitum through the central water supply, in accordance with state regulations: SanPiN 2.1.3684-21, SanPiN 1.2.3685-21, Rospotrebnadzor, Russian Federation. Water samples were periodically analysed for contamination.

The animal’s diet was balanced for proteins, fats, vitamins, and carbohydrates. In terms of the composition, the feed contained dietary fibre, consisted of complete feed, fruits, vegetables, rice porridge with raisins, biscuits, briquetted feed according to the average feed intake standards. The animals were fed three times a day: 8:00–9:00—granulated balanced food; 11:00–12:00—juicy food (fruits, vegetables), rice porridge, cookies; from 14:00 to 15:00 granulated balanced food (according to local regulations: SOP AC 11 Feeding Individually Caged Monkeys).

The experiments were approved by the bioethical committee of the Research Institute of Medical Primatology (protocol number 83, dated 18 March 2022) and were carried out in accordance with the International Association for Evaluation and Accreditation of the Care of Laboratory Animals. All macaques included in the study were found to be healthy based on regular veterinary examination and tests for tuberculosis and herpes virus B and according to previous records.

### 2.2. Blood Sampling and Analysis

Before blood sampling, the animals did not receive food for 12 h. To take a blood sample, an experienced animal technician carefully immobilized the conscious monkey, and a qualified veterinarian took 4 mL of blood from the inguinal vein with a syringe (21 G needle) and immediately poured into tubes with potassium EDTA. All sample was analysed with an automatic haematology analyser MEK-7300K.

For the flow cytometry analysis, an aliquot of whole blood was stained with an antibody cocktail, lysed with the FarmLyse buffer (BD), washed twice with the BSA Stain Buffer (BD) and analysed on a flow cytometer. Three antibody cocktails were used for different purposes. Cocktail A, that included antibodies to CD3 PerCP-Cy5.5, CD4 FITC, CD8 PE and CD45 APC, was designed to detect T cell populations. Cocktail B included antibodies to CD20 FITC, CD3 PerCP-Cy5.5, CD16 PE, CD56 PE and CD45 APC. It was used to analyse B and NK cells. The samples stained either with Cocktail A or Cocktail B were analysed with a BD FACS ACCURI C6+ flow cytometer in the Research Institute of Medical Primatology. To study the CD16 and CD56 distribution in lymphocytes a modified Cocktail B was used with CD56 PE substituted for CD56 BV786 Clone NCAM16.2 (for more information see Supplementary Table S1). These samples were stained according to the same protocol and analysed with BD LSRFortessa in the Core Facility for Cell Technologies and Immunology of the Sirius University (Sochi, Russia). The flow cytometer was equipped with 4 lasers (405, 488, 561 and 640 nm) and appropriate detectors for the used fluorochromes.

All flow cytometer reagents were purchased from BD Biosciences. Cell population analysis was performed using the FlowJo v10.8 Software (BD Life Sciences). UMAP analysis was carried out in a FlowJo plugin UMAP v3.1 [13].

We analysed 28 blood populations using flow cytometry and 25 blood parameters using a haemoanalyser. The study involved 63 cynomolgus monkeys to establish baseline standards for young monkeys (3–7 years old) and very old monkeys (17–31 years old). No animals were euthanized in this study, and they continued to live in the apery of the Institute of Medical Primatology to the present day.

### 2.3. Statistical Analysis

For all data in groups, cell populations were presented as mean ± standard deviation (Supplementary Table S1). Pearson correlation analysis between all individual parameters, all data processing, statistical analysis, and visualization were performed using GraphPad Prism version 9.5.0 for Windows (GraphPad Software, (Dotmatics) Boston, MA, 02110, USA). Algorithm of data analysis in GraphPad: (1) detection of outliers with the ROUT method (Q = 1%), (2) Shapiro-Wilk test for normal (Gaussian) distribution of the cleaned data, (3) pairwise group comparison with either unpaired t-test (two-tailed, *p* < 0.05) for normal distributed data or Mann-Whitney test (two-tailed, *p* < 0.05), for non-Gaussian distributed data. Level of difference significance in the text and figures were indicated as: not significant - ns, (*p* > 0.05), * (*p* ≤ 0.05), ** (*p* ≤ 0.01), *** (*p* ≤ 0.001), **** (*p* ≤ 0.0001).

Data significantly different in at least one group were normalized and visualized by the cluster analysis using the Pearson correlation method and CIMminer web-based software: https://discover.nci.nih.gov/cimminer/oneMatrix.do (accessed on 12 January 2023).

## 3. Results

**Figure 2:**
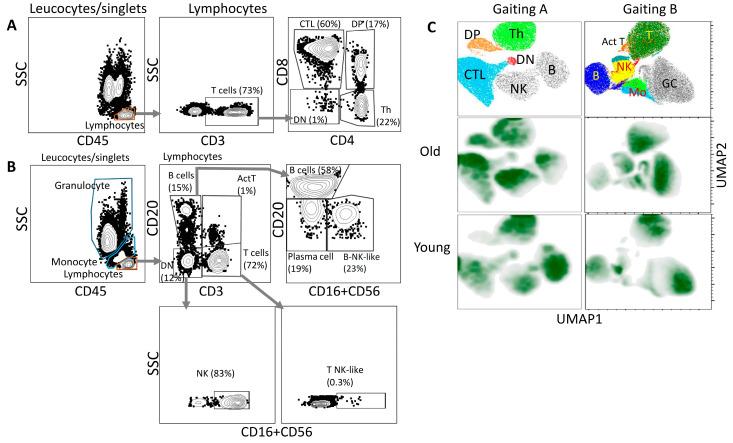
Gating strategy and uMAP clustering of blood cell populations distribution for young (3–7 years old) and old (17–31) monkey group. T-cell population gating strategy (**A**) and B/NK-cells populations gating strategy (**B**) are shown. Comparative analysis old and young group of monkeys are visualized for T cell populations Gating A and for B/NK-cells populations Gating B (**C**).

The flow cytometry analysis was performed with antibody cocktails that allow distinguishing of major subtypes of lymphocytes either T-cells (T-helpers, CTLs, double positive CD4+CD8+, Cocktail A) or (B, T, NK-cells, Cocktail B, see Methods for details). Figure 2 shows the gating strategy for the analysis of T-cells (Figure 2A) and for the analysis of B-, NK-cells (Figure 2B). Population percentages for animals of different ages are presented in Supplementary Table S1. Projection of the manually gated populations on UMAP graphs for old and young C. monkeys groups reveals age related dynamic changes in the distribution of leucocytes (Figure 2C). Comparison of two age groups (Cocktail A/Gating A) showed that the number of CD3+CD4+CD8+ cells—double positive population (DP) and the cluster of CD3+CD4-CD8+ cells (CTL) increased and CD3+CD4+CD8-cells (Th) cluster decreases in old monkeys (Figure 2C, left panel). Analysis of B/NK cells (Cocktail B/Gating B) shows an increase in the number of monocytes, as well as a decrease in CD20+CD3+ (Act T). The latter contain a population of activated T cells (Figure 2C, right panel).

**Figure 3:**
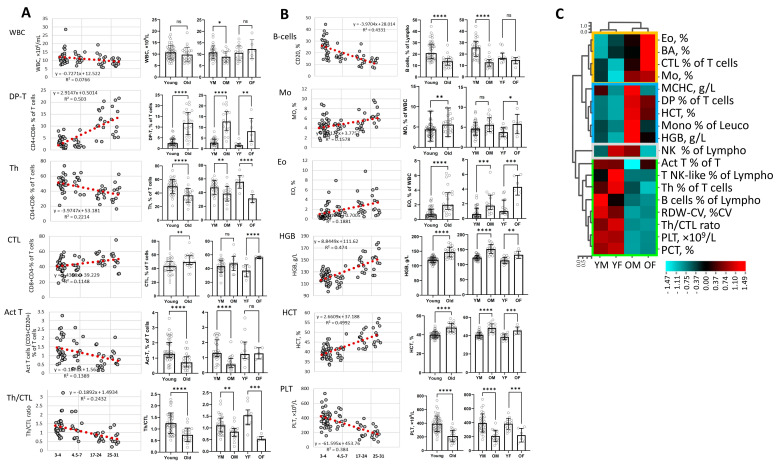
Age related changes in the blood population. The graph displays regression models showing the relation of selected blood populations to animals age (**A**,**B** left plots). Regression equation and correlation with the model is shown on each plot. Young and Old animals were presented either by age all together or by age and sex (**A**,**B** right plots). Young males (YM) and young females (YF) were included in the sample of 3–7 years old, old males (OM) and old females (OF) were included in the sample of 17–31 years old (**A**,**B** right plots). Cluster analysis is presented for all populations that have a significant difference between any two age groups. Colour coding scale shows correlation coefficient (r) level (**C**). Population name (abbreviation) and explanation—see in the text. Levels of difference significance were indicated as: not significant - ns, (*p* > 0.05), * (*p* ≤ 0.05), ** (*p* ≤ 0.01), *** (*p* ≤ 0.001), **** (*p* ≤ 0.0001).

Just as in the granulocyte population, additional subpopulations appeared with age, which could not be identified using markers form cocktail B. This requires further selection of NAP-specific antibodies. The isolated populations were analysed after further division into groups to construct predictive regression equations (Figure 3A,B). The analysis showed that the number of white blood cells (WBC) remained stable with age and even in older animals it fluctuates around 10,000/uL (Figure 3A, WBC). While the WBC count remained stable, the percentage of the CD3+CD4+CD8+ T cell population (DP-T) increases with age. A moderate correlation (r = 0.5, *p* = 0.02) of the experimental data was found with a linear regression model predicting the age dependence of the DP-T population. (Figure 3A, DP-T). Significant increase in the percentage of DP-T cells was observed both in males and females in the 17–31 years old group (*p* < 0.0001 and *p* < 0.01) (Figure 3A, DP-T—right plot).

There was a downward trend in CD3+CD4+CD8- T-helpers (Th) in males while significant decrease was found only in old females (Figure 3A. Th). In macaque females, the percentage of CD3+CD4-CD8+ cells containing predominantly a population of cytotoxic lymphocytes (CTL) increased significantly. Accordingly, the Th/CTL index drastically decreased with age both in females and to a lesser extent in males (Figure 3A. Th/CTL right plot). Note, Th/CTL index proved to be a more reliable indicator of human age.

The analysis has shown that an increase in the number of monocytes (Mo) and eosinophils (Eo) with age has a stable trend. However, significant increase was only found in the number of eosinophils in older females compared to younger ones (Figure 3B, EO).

Analysis of the haemoanalyser data revealed a trend towards a decrease in erythrocyte volume variation (RDW-CV) and a reduction in platelet count (PLT), which was significant for both sexes (*p* < 0.05). There was also a trend towards an increase in haematocrit (HCT) and haemoglobin concentration (HGB) with age. Significant correlations (r = 0.5; *p* < 0.05) with a linear increase-with-age regression model for HGB and HCT and correlation with a negative linear regression for PLT and RDW-CV (r = 0.4 and r = 0.5 *p* < 0.05) showed the possible predictive value of these indicators for the analysis of the immunological age and health of macaques.

To generalise the obtained data on cell populations that change with age, we applied clustering using the Pearson correlation method. The analysis included those populations in which at least one of the groups showed a significant change with age. Four clusters were identified, the first one included a group of cell populations EO, BA, CTL, MO, which increased with age mainly in old females (OF), (Figure 3C, yellow frame).

The second cluster (blue frame) shows a predominant growth in males in such indicators of erythropoiesis as MCHC, HGB, HCT, and the percentage of DP population of T cells. It is worth noting that in this cluster, DP population and HCT are increased in both older males and females. The third cluster (light-grey frame) demonstrates multidirectional dynamics of NK cells. In males, the percentage of NK cells rises with age, while in females, on the contrary, it reduces. The fourth cluster mainly represents cell populations that decrease in number with age. These are lymphocytes including T-helpers (Th), B-cells and Th/CTL ratio. The cluster also includes minor populations—activated T cells (Act T), T NK-like. In addition, the cluster includes two indicators for assessing the number of platelets (PLT) and PCT, % of platelets, both indicators decrease with age, but they correlate with each other. At the same time, there is a decline in RDW-CV percent (erythrocyte width variation coefficient).

**Figure 4:**
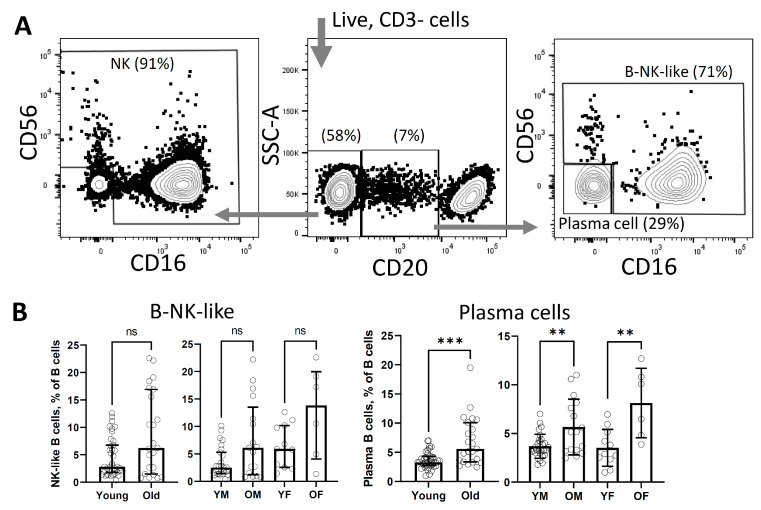
Age-related changes in the population of B- and NK-cells. Gating strategy for CD3-negative lymphocyte population (**A**). Age differences in macaques for the CD20lo population (**B**). Levels of difference significance were indicated as: not significant - ns, (*p* > 0.05), ** (*p* ≤ 0.01), *** (*p* ≤ 0.001).

In addition, we analysed the CD3-CD20lo population, which was approximately 70% positive for CD16 and CD56. To analysed the contribution of each marker separately, an additional multicolour analysis was performed (Figure 4A, middle and right plot). CD3-CD20loCD16+ and CD56+ were found to be similar to the NK cell population (Figure 4A, left and right plot). As a result, significant differences with the age of both sexes were found only in CD3-CD20loCD16-CD56- plasma cells. (Figure 4B).

## 4. Discussion

The cynomolgus macaque is one of the evolutionary closest to human non-anthropoid primate species, and a valuable animal model increasingly used for preclinical, immunological, behavioural, and other studies.

The immune system of captive monkeys can differ significantly from wild animals or from monkeys kept in other aperies due to objectively diverse living conditions in different animal facilities. In this study, the animals included in the sample belonged to the 3rd generation living in captivity. The presented data demonstrated the stability of the blood parameters of cynomolgus macaque of various origins and introduced additional statistical data on the age and sex of the animals. Most haemoanalyser data for young animals were within the range of variation reported in previously published studies [6,14,15]. A comparative analysis of these data with other publications can only be carried out for relatively young animals, since there are practically no studies on very old animals. To our best knowledge, very old cynomolgus monkeys (17–24 and 25–31 years old) have not been previously studied, which makes this research unique. In addition, both males and females were represented in the sample, which allowed to examine sex differences in the composition of immune cells in very old animals. This investigation is essential for assessing the results of preclinical studies, for sampling healthy animals, for the analysis of novel the medications against senile diseases and age-related pathologies of the immune system.

Analysis of white blood cells (WBC) with a haemoanalyser showed that WBC number was stable throughout the life of primates. Human WBCs are usually in the range 5000 to 11,000/mL. The number of WBC in C. monkeys remains on average at ~10,000/mL throughout life, which indicated a good state of the immune system of animals in this sample, the absence of anaemia (when < 5000/mL) or other pathologies of haematopoiesis. Only one young animal showed a significant excess of the WBC count range. (Figure 3A, WBC) [16]. The stable WBC count in the ontogeny of C. monkeys allowed using the percentage of cell populations as a basis for analysis.

In line with previous observation, percentage of CD4+CD8+ double positive (DP) population of T-cells increased with age [17,18]. In humans, the DP population increases in the blood during normal aging, or during inflammatory, autoimmune [19,20] or oncological diseases [21,22]. It is assumed that during aging this increase may be associated with the degradation of thymus function and the release of these cells into the periphery. In healthy young animals, DP population is present in the thymus but not in the peripheral blood. It has been shown that the increase in this population in blood begins as early as 7 years of age, although the thymus involution in macaque occurs at about 11 years of age [23].

For thymic involution analysis, the count of coding joint of T-cell receptor excision circles (cjTREC) in T-cells is widely used. Indeed, the decrease in the level of cjTREC in T cells was significant among the group of seven-year-old animals, which differed in the number of DP cells in the blood [23]. In this study, no increase in DP population was found in the 4–7-year-old group while in the 17–24-year-old group, a significant elevation in the DP cell number was observed. Nevertheless, even at the age of 24–31 there was a substantial variation in the number of DP cells that cannot be explained by thymic involution alone. By the age of 17, thymic involution should have ended in the vast majority of animals. Yet, in some C. monkeys, the level of DP cells remained at the level of young (Figure 3A DP-T). Recent studies have found significant heterogeneity in the DP cell population. 12 new subpopulations in DP cell population of a 10-year-old *Macaca fascicularis* using single cell RNA sequencing were discovered [17]. These data have been confirmed on 6-years-old rhesus monkey (*Macaca mulatta*) [18]. This heterogeneity may result in one or more populations being predominantly overgrown with age due to its disease history or other reasons. The variation in DP cells in a very old group of macaques requires further studies to determine heterogeneity compared to animals of an average age (6–10 years).

We also noted a trend towards a reduction in the number of CD3+CD20+ cells (Act T) indicating a general decrease in the functional ability of activated T-cells. These cells produce high levels of IL-17A and/or IFN-γ and play role in cancer and HIV infection [24]. The significant decline in Act T cells only in male macaques likely points to the well-documented evidence in humans of a more rapid decline in immune surveillance in older men compared to women [25,26].

In humans, a decrease in peripheral blood in the amount of both CD3+CD4+ (Th) and CD3+CD8+ (CTL) in old age was described [27]. In contrast to humans, both male and female C. monkeys showed an increase in CTL in the old group, while the amount of Th decreases in the same way as in humans. Typically, in human the CD4/CD8 ratio is used as an index of health and aging. At a young age, the index varies around 2.0, and in old age it may decrease below 1.0 [28]. Simultaneous increases in Th and CTL have been noted in inflammatory diseases and autoimmune disorders [29,30]. In this study, *Macaca fascicularis* showed a decrease in Th and an increase in CTL. In the young group Th/CTL index exceeded 1 in more than 80% of animals, and in the group of elderly animals the index fell below 1 in more than 80% animals (Figure 3A., Th/CTL).

Several studies have shown that differences in the immune system of males and females are associated with age-related difference in level of sex hormones [31]. Significantly more CTL cells have been documented in men than in women [32]. The same trend was noted in male macaques, however, in older animals, the CTL increased more in females than in males (Figure 3A).

It is well known that oestradiol (E2) plays a critical role in immune function. A decrease in the level of E2 in the blood reduces both the number of CD4+ cells and the ratio of CD4/CD8 [33]. As in humans, in young C. monkeys, the Th count and the CD4/CD8 cell ratio are also higher in females than in males [32,34]. In older animals, the percentage of CD4+ cells and Th/CTL index decreased more in females than in males (Figure 3A). The decrease in Th/CTL index was significant both in females and males, which specified considerable health deterioration with age.

The effect of E2 on the proliferation of B-cells was also shown [35]. In women, there was an increase in the number of B-cells with age due to age-associated B-cells (ABCs) expansion, although the number of B-cells producing mature antibodies is reduced in both men and women [36]. In older male macaques, there was a significant decrease in the number of B-cells compared to younger ones. Young females have fewer B cells than males and their number does not change between age groups (Figure 3B). Further blood studies of sex hormones, ABC cells, and senescent cells will suggest the reasons for the stabilization of the number of B-cells in old females and the fall in their number in males.

In further analysis, a new population of CD3-CD20loCD16+/CD56+ was found (Figure 2B, B-NK-like). Since this population consisted of cells expressing CD20 at medium and low levels, we initially classified the cells as plasmablasts or plasma cells [37]. Analysis of publications showed that neither human plasmablasts nor plasma cells carry the CD16+ or CD56+ markers [38]. Additional multi-channel FACS analysis allowed us to classify CD20loCD16+/CD56+ cells as previously undescribed CD20lo NK-cells due to the similarity of CD16 and CD56 expression patterns with NK-cells (Figure 4A, compare left and right plots). While populations of CD3-CD20loCD16-CD56- we referred to plasmablasts or plasma cells. Previously, CD20 expression was reported on NK lymphoma but not on normal NK-cells [39]. Analysis of these novel population have shown that the number of CD3-CD20loCD16-CD56- plasmablasts/plasma cells increases significantly with age in both females and males (Figure 4B, plasma cells), while in the population of CD3-CD20loCD16+/CD56+ (Figure 4B, B-NK-like) age growth remained at the trend level.

The number of erythrocytes, as well as the amount of haemoglobin and haematocrit, had a steady tendency to increase with age. No anaemic animals with HGB levels less than 110 g/l were found (Figure 3B,C). In humans, there is a slight increase in haemoglobin levels in old age. In addition, about 18% develop anaemia after age 80. The authors suggest that control of haemoglobin levels with either administration of erythropoietin or haematinics improves functional status and survival in people over 80 [40]. In older C. monkeys, a significant (two-fold) decrease in platelets was found. In humans, platelet counts have also been shown to more than halve after age 70 (~20 years in macaques). If at the age of 20–60 the average platelet level was 20 × 10^3^ platelets/μL, then after 70 years it was about 10 × 10^3^ platelets/μL [16].

Studies of the dynamics of cell populations provide important information about the functional changes in immunity. Building predictive models using cell population dynamics provides an additional dimension for quantifying the biological and immunological age of humans and animals [41,42]. This is especially important for preclinical studies to analyse the impact of medicinal substances on the immune system and biological age of animals, to determine health indicators and age-related changes in NAPs [43,44]. To build such models, we applied regression analysis. As a result, the strongest correlations with calendar age regression models with immune populations were found for DP-T (R^2^ = 0.50 *p* < 0.05), for B-cells (R^2^ = 0.43 *p* < 0.05 for males), HGB (R^2^ = 0.47 *p* < 0.05), HCT (R^2^ = 0.50 *p* < 0.05) and platelets (R^2^ = 0.38 not significant) (Figure 3A,B). Further analysis of additional populations and inflammatory factors may help build a model of immunological aging in non-anthropoid primates [2,44].

Cluster analysis showed that the increase in the number of myeloid cells forming a cluster of EO, BA, Mo was more pronounced in females. The same cluster included the amount of CTLs, which also significantly rose in females (Figure 3C, yellow frame). High level of progesterone can mediate increase eosinophils number in human [45]. The increase in EO level observed in young and old females compared with males (Figure 3B,C). Perhaps, differences in the level of sex hormones in macaques form such a correlation cluster of cells even in old age when the level of sex hormones is reduced. What mechanism underlies the predominant boost in other populations included in this cluster remains unclear.

The next cluster characterized the predominant increase in erythroid parameters (HGB, HCT, MCHC), and percentage of monocytes in the elderly C. monkey males. In humans, differences in haemoglobin levels and, to a lesser extent, haematocrit between the sexes have also been associated with sex hormones [46], however, such differences between the sexes in old macaques were observed for the first time [47]. This cluster also contained cells that were enlarged with age in the entire old population and were not entire associated with sex, such as a double positive population of T-lymphocytes (DP-T) and haematocrit (HCT) (Figure 3C, blue frame).

The NK-cell cluster showed the greatest sex differences (grey frame), increasing in males with age and decreasing in females (Figure 3C). In contrary, in humans, a reduction in CD56+ NK cells with age were shown [48]. The accumulation of NK-cells is on average higher in men than in women [49]. In current studies, the same trend was noted, but only in older male macaques.

And the last cluster included cells that declined with age in *Macaca fascicularis* (Green). The cluster included minor populations Act T, T/NK-like, and major populations of B-cells, T-helpers, two platelet parameters (PLT-count, PCT). This cluster also included the main indicator of the decline in the function of the immune system Th/CTL ratio. This cluster appears to reflect a general decline in the adaptive immune response in aged macaques [44].

## 5. Conclusions

Thus, we found the following age-related changes in the haematopoiesis of *Macaca fascicularis*. An increase in myeloid cells was noted, including Mo (in females to a greater extent than in males) and Eo in both sexes. Erythroid components increased with age to a greater extent in males than in females. These changes probably indicated an increase in the age-related inflammatory response and were associated with the well-documented myeloid-erythroid bias of hematopoietic stem cells.

The general senile deterioration in the function of the adaptive immune system was characteristic of both sexes, but for each in its own way. The decrease in the Th/CTL ratio, the number of Th cells and the increase in CTL were more significant in females, while the decrease in the number of ActT and B-cells was observed only in males. For both sexes, there was an increase in the percentage of DP cells, plasma cells and loss of platelets. The overall decrease in the number of platelets was accompanied by an increase in the amount of haemoglobin, haematocrit, and erythrocytes, which may indicate an increase in capillary thrombosis, microhaemorrhage, and background inflammation in aging primates.

The additional CD3-CD56+CD20lo NK-like population found in this study had only a non-significant tendency to increase with age.

Analysis of these cell populations has highlighted immune cells predicting the age of animals. These populations included DP-T, HCT and HGB. All these populations were highly correlated (R^2^ ~ 0.5) with the linear regression models. Other populations had sex-dependent predictivity. The level of B-cells predicted only for males and the level of CTL for females only. The generally accepted indicators of health Th/CTL, the number of Th, Mo, Eo, had weak predictivity in small and medium samples due to the strong variability of these populations in *Macaca fascicularis*.

## Data Availability

All data presented in this manuscript, including supplementary materials. Other primary data, such as flow cytometry and haemoanalyser files or other manual entries in workbooks, are available on request and in accordance with the Federal Law (Russian Federation of 27 July 2006 N 152-FZ).

## Supplementary Materials

The following supporting information can be downloaded at: https://www.mdpi.com/article/10.3390/life13020316/s1, Supplementary Table S1: List of antibodies used in the study and primary data from flow cytometry and hemoanalyzer.

## Abbreviations

Haemoanalyser populations	
EO	percentage of eosinophils in the blood
BA	Basophils
Mo	Monocytes
PLT × 10^9^/L	Platelets
PCT, %	percentage of platelets in the blood
HCT, %	Haematocrit
HGB, g/L	Haemoglobin
MCHC g/L index	concentration of haemoglobin in a single erythrocyte
RDW-CV, %	Erythrocyte width variation coefficient
Flow cytometry populations	
CTL % of T-cells	percentage of CD4-CD8+ cells (Cytotoxic Lymphocytes) in CD3+ cell population
DP % of T-cells	percentage of Double Positive CD4+CD8+ cells in CD3+ cell population
Th, % of T-cells	percentage of CD4+CD8- cells (T-helpers) in CD3+ cell population
Act T, %	percentage of CD3+CD20+ cells (activated T-cells) in CD3+ cell population
Th/CTL index	CD3+CD4-CD8+/CD3+CD4+CD8- ratio
T NK-like, %	percentage of CD3+CD20-CD16&CD56+ cells in lymphocyte gate
B, %	percentage of CD20+CD3-CD16-CD56- cells in lymphocyte gate
B-NK-like, %	percentage of CD20loCD3-CD16&CD56+ cells in lymphocyte gate
Plasma cells, %	percentage of CD20loCD3-CD16-CD56- cells in lymphocyte gate
